# Thymic Stromal Lymphopoietin Interferes with the Apoptosis of Human Skin Mast Cells by a Dual Strategy Involving STAT5/Mcl-1 and JNK/Bcl-x_L_

**DOI:** 10.3390/cells8080829

**Published:** 2019-08-05

**Authors:** Tarek Hazzan, Jürgen Eberle, Margitta Worm, Magda Babina

**Affiliations:** Department of Dermatology, Venerology and Allergy, Charité—Universitätsmedizin Berlin, Charitéplatz 1, 10117 Berlin, Germany

**Keywords:** mast cells, TSLP, skin, apoptosis, survival, STAT5, JNK, Mcl-1, Bcl-x_L_, RNA interference

## Abstract

Mast cells (MCs) play critical roles in allergic and inflammatory reactions and contribute to multiple pathologies in the skin, in which they show increased numbers, which frequently correlates with severity. It remains ill-defined how MC accumulation is established by the cutaneous microenvironment, in part because research on human MCs rarely employs MCs matured in the tissue, and extrapolations from other MC subsets have limitations, considering the high level of MC heterogeneity. Thymic stromal lymphopoietin (TSLP)—released by epithelial cells, like keratinocytes, following disturbed homeostasis and inflammation—has attracted much attention, but its impact on skin MCs remains undefined, despite the vast expression of the TSLP receptor by these cells. Using several methods, each detecting a distinct component of the apoptotic process (membrane alterations, DNA degradation, and caspase-3 activity)*,* our study pinpoints TSLP as a novel survival factor of dermal MCs. TSLP confers apoptosis resistance via concomitant activation of the TSLP/ signal transducer and activator of transcription (STAT)-5 / myeloid cell leukemia (Mcl)-1 route and a newly uncovered TSLP/ c-Jun-N-terminal kinase (JNK)/ B-cell lymphoma (Bcl)-x_L_ axis, as evidenced by RNA interference and pharmacological inhibition. Our findings highlight the potential contribution of TSLP to the MC supportive niche of the skin and, vice versa, highlight MCs as crucial responders to TSLP in the context of TSLP-driven disorders.

## 1. Introduction

Mast cells (MCs) are tissue-resident key effector cells of Immunoglobulin E (IgE)-mediated allergic and inflammatory responses, including common skin disorders, such as atopic dermatitis (AD) and psoriasis [1,2,3,4,5,6]. As hematopoietic cells, MCs complete their differentiation into mature subsets only after arriving in peripheral organs, such as the skin, lung, and gut. The skin is the tissue with the greatest MC density, even in the steady-state [7], which can further increase in the context of cutaneous inflammation, a process accompanied by an increased abundance of pro-inflammatory mediators [2,4,8]. MC maintenance under both physiological and pathological conditions requires constant signals from the micro-environment [9,10,11]. In the skin, the stem cell factor (SCF)/KIT (CD117) axis is believed to have a critical impact on MC survival, but other factors are likely to contribute, although their relevance has remained less defined. In fact, MCs are characterized by excellent survival properties in their natural skin habitat [12,13]. This property is maintained ex vivo, as skin MCs show remarkable persistence, even in the absence of SCF. Accordingly, several studies have indicated that the protection from cell death requires factors other than SCF [14,15,16,17].

Thymic stromal lymphopoietin (TSLP) is mostly expressed by epithelial cells, including keratinocytes [18,19,20]. The cytokine has attracted considerable attention, as it is renowned for its Th2 skewing potential and is implicated in widespread allergic and inflammatory disorders in the skin and lung [18,19,20,21]. As a consequence, TSLP targeting by means of tezepelumab (anti-TSLP antibody) is in clinical trials for asthma and atopic dermatitis [22,23].

While the responsiveness of MCs to TSLP has been occasionally reported [24,25,26,27,28], it remains to be established if and how MCs of human skin origin are targeted by TSLP. This is of relevance because MCs are known for their highly heterogeneous nature [7,29,30], so that their response pattern will depend on the precise MC subset and location.

Here, we identify TSLP as a pro-survival factor of human skin MCs. We kinetically resolve the sequence of events underlying TSLP’s antiapoptotic action. Initially, TSLP activates the signal transducer and activator of transcription (STAT)-5 and c-Jun-N-terminal kinase (JNK), which are both implicated in survival prolongation. STAT5 acts by enhancing myeloid cell leukemia (Mcl)-1, while JNK is responsible for B-cell lymphoma (Bcl)-x_L_ upregulation. These two well-known antiapoptotic Bcl-2 proteins jointly organize the survival of skin MCs.

## 2. Materials and Methods

### 2.1. Purification and Culture of Human Skin Mast Cells and Other Skin Cells

MCs were isolated from human foreskins, whereby material from several donors (typically between 2 and 9) was combined for one experiment to achieve the cell numbers required, as described [31,32,33]. The skin was obtained from circumcisions, with the informed consent of the patients or legal guardians and approval by the university ethics committee. The experiments were conducted according to the Declaration of Helsinki Principles. The purification was performed using an optimized and frequently employed protocol, as described [34,35], with several modifications. Briefly, human skin was cut into strips and treated with dispase (BD Biosciences, Heidelberg, Germany) at 3.5 U/mL and 4 °C overnight. The epidermis was removed, and the dermis was chopped into small pieces and digested with 1.5 mg/mL collagenase type 1 (Cat.Nr. LS004197; Worthington, Lakewood, NJ, USA), 0.75 mg/mL hyaluronidase type 1-S (Cat.Nr. H3506-5G, Sigma, Deisenhofen, Germany), and DNase I at 10 μg/mL (Roche, Basel, Switzerland) at 37 °C in a shaking water bath for 1.15 h. The cells were filtered from remaining tissue. MC purification was achieved with anti-human c-Kit microbeads and the auto-magnetic activated cell Sorting (MACS) separation device (both from Miltenyi-Biotec, Bergisch Gladbach, Germany). MC purity consistently exceeded 98%, as assessed by acidic toluidine-blue staining (0.1% in 0.5 N HCl) and by KIT/FcεRI double staining [34,36]. Viability by trypan blue exclusion was >99%.

Skin MCs were used either ex vivo or cultured in Basal Iscove medium, supplemented with 10% fetal calf serum (FCS, Biochrom), SCF (Peprotech, Rocky Hill, NJ, USA) (at 100 ng/mL), and interleukin (IL)-4 (Peprotech) (10 ng/mL) [37,38,39].

Skin fibroblasts and keratinocytes, used as controls, were isolated according to routine protocols [40,41]

### 2.2. Cell Treatment

Skin MCs were treated with different concentrations of TSLP (Peprotech) for the indicated times in minimal medium, consisting of Basal Iscove medium (with stable glutamine; Biochrom, Berlin, Germany), supplemented with 0.5% bovine serum albumin (BSA) (Serva, Heidelberg, Germany). After incubation, cells were processed for downstream applications (as described below).

For kinase inhibition studies, cells (at 5 × 10^5^ cells/mL) were pre-incubated for 15 min, with pimozide (STAT5 inhibitor, 5 µM), SCH772984 (extracellular signal–regulated kinases (ERK)1/2 inhibitor, 5 µM) (both from Selleckchem, Houston, TX, USA), SB203580 (p38 inhibitor, 5 µM), or SP600125 (JNK inhibitor, 5 µM) (both from ApexBio, Houston, TX, USA), prior to the addition of TSLP. After incubation, cells were processed for downstream applications (see reverse transcription - quantitative polymerase chain reaction (RT-qPCR), immunoblotting, and analysis of survival).

### 2.3. Analysis of Survival

MCs (at 5 × 10^5^ cells/mL) were treated with different concentrations of TSLP in minimal medium for the indicated times and processed for survival detection by different methods, based on our previously established protocols [14].

For YoPro^TM^-1 staining, cells were incubated with the YoPro^TM^-1 dye (Thermo Fisher Science, Berlin, Germany) and propidium iodide (PI, BD Biosciences) for 30 min on ice. YoPro^TM^-1 staining detects membrane porosity and, combined with PI, can serve to distinguish apoptotic cells from viable and necrotic cells.

Apoptosis detection by annexin V-fluorescein isothiocyanate (FITC)/PI (BD Biosciences) was performed according to the protocol of the supplier. In brief, cells were stained with annexin V-FITC and PI in annexin V binding buffer (BD Biosciences) for 20 min at room temperature (RT). Annexin V-FITC detects the externalization of phosphatidylserine and, combined with PI, can distinguish apoptotic from viable and necrotic cells.

**Figure 1:**
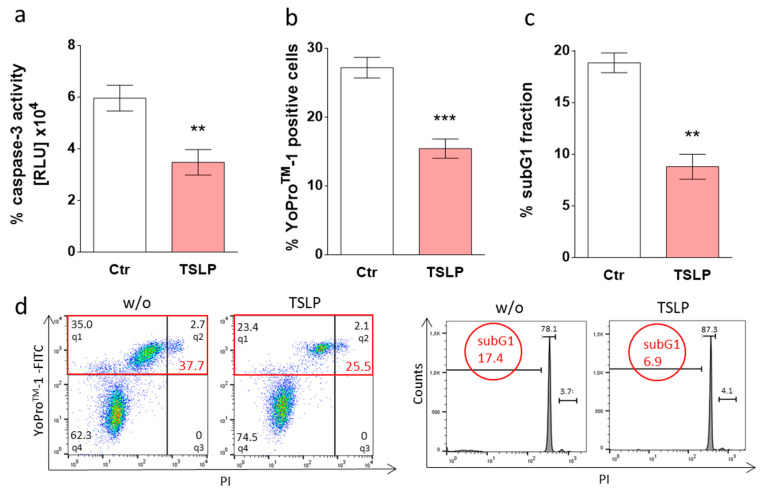
Thymic stromal lymphopoietin (TSLP) counters apoptosis of skin mast cells (MCs) upon grow factor (GF) withdrawal. Skin-derived MCs were kept without or with TSLP (at 7.5 ng/mL) in serum/GF-free medium. (**a**) Caspase-3 activity as determined after 24 h by Caspase-Glo^®^ 3/7 assay; the results represent the mean ± SEM of six independent experiments. RLU = relative luminescence units (×10^4^). (**b**,**c**) Percentage of cells with (**b**) YoPro^TM^-1 positivity (corresponding to the percentage of early and late apoptotic/necrotic cells combined) after 24 h or (**c**) fragmented DNA (propidium iodide (PI) staining, corresponding to the percentage of subG1 positive cells) after 48 h. The results represent the mean ± SEM of six (YoPro^TM^-1) or four (PI) independent experiments. (**d**) Representative flow cytometry dot plots/histograms of (**b**) (specified in red is the percentage of early and late apoptotic/necrotic cells combined) and (**c**). The data were analyzed by paired *t*-test, ** *p* < 0.01, *** *p* < 0.001. *q*—quadrant.

For YoPro^TM^-1/PI and annexin V-FITC//PI, double-positive cells were considered cells in advanced apoptosis or necrosis, whereas YoPro^TM^-1- or annexin V-FITC-positive but PI-negative cells were considered early apoptotic. Double-negativity was indicative of viable cells. YoPro^TM^-1 or annexin V-FITC positivity was calculated as the sum of the percentages displayed in quadrant (q)1 and q2, based on the values given in Figure 1b,c and Figure S2, with early-apoptotic, late-apoptotic/necrotic, PI-single positive necrotic, and viable cells being shown in quadrant (q)1, q2, q3, q4, respectively.

For PI staining, cells were treated with PI (Sigma–Aldrich, Taufkirchen, Germany) for 1 h at 4 °C, which was performed to detect the percentage of hypodiploid nuclei corresponding to cells with fragmented DNA. The cells were measured on a MACS Quant FACS (fluorescence-activated cell sorter) (Miltenyi Biotec, Bergisch Gladbach, Germany) and analyzed using the FlowJo software (FlowJo LLC, Ashland, OR, USA).

The caspase-3 activity of MCs was measured using a luminometric assay kit (Caspase-Glo^®^ 3/7; Promega, Mannheim, Germany), providing a proluminescent caspase-3/7 substrate, which is cleaved to release luminescence, which was detected by means of a microplate reader (Perkin Elmer, Berlin, Germany), as described [14].

Prior to perturbation experiments, kinetics series were performed to identify the shortest incubation period, after which survival promotion by TSLP (by YoPro^TM^-1 and caspase-3) could be unambiguously detected (not depicted). This served to ensure a predominance of direct effects and limit secondary effects (potentially resulting from other effects of the inhibitors/knockdown strategies on diverse cellular pathways). We found this period to be 8 h.

Using this period, the ratio of YoPro^TM^-1 positivity (1) or caspase-3 activity (2) in TSLP-treated versus untreated MCs was calculated by the following formulas in perturbation experiments. Based on the values given in Figures 3 and 5a, with early-apoptotic, late-apoptotic/necrotic, PI-single positive necrotic, and viable cells being found in q1, q2, q3, and q4, respectively, the calculation was as follows:

% Rescue effect of TSLP =

(1) $$\left(100\times \frac{{\left[q1+q2\right]}_{\mathrm{untreated}}}{{\left[q1+q2\right]}_{\mathrm{treated}}}\right)-100,$$

(2) $$\left(100\times \frac{{\mathrm{RLU}}_{\mathrm{untreated}}}{{\mathrm{RLU}}_{\mathrm{treated}}}\right)-100.$$

### 2.4. Staining of Intracellular and Extracellular Proteins

For the intracellular staining of signaling intermediates, skin MCs (5 × 10^5^ cells/mL) were deprived of GF (growth factors)/serum for 16 h in minimal medium. After starvation, cells were incubated for a further 15 min with TSLP, then fixed with 4% paraformaldehyde (PFA) for 30 min at RT and permeabilized with 0.1% Saponin for 20 min at 4 °C. Cells were stained with anti-pSTAT5 or anti-pJNK primary antibodies (each diluted 1:400, both from Cell Signaling Technologies, Danvers, MA, USA) (or the respective isotype control) for 30 min on ice, followed by incubation with Phycoerythrin (PE)-labeled secondary antibodies (Jackson Immunoresearch, Cambridgeshire, UK) for 30 min on ice.

For the detection of the TSLP receptor, flow cytometry was performed according to routine protocols [42]. Briefly, MCs were blocked for 15 min at 4 °C with human AB-serum (derived from patients with blood group AB) (Biotest, Dreieich, Germany) and incubated with an anti-human TSLP-Receptor PE-conjugated antibody (Immunotools, Friesoythe, Germany) for 30 min at 4 °C.

Cells were measured on a MACS Quant FACS (Miltenyi Biotec) and analyzed using the FlowJo software (FlowJo LLC). Data were shown as net mean fluorescence intensity (MFI), as calculated by the following equation:

(3) $$\mathrm{net}\;\mathrm{MFI}={\mathrm{MFI}}_{\mathrm{target}}-{\mathrm{MFI}}_{\mathrm{Isotype}\;\mathrm{control}}.$$

### 2.5. siRNA Transfection

RNA interference in MCs was performed according to a recently established protocol [36], using the Accell^®^ siRNA transfection technology (GE Healthcare Dharmacon, Lafayette, CO, USA). MCs were transfected by gene-targeting siRNA or non-targeting siRNA (serving as control). Briefly, MCs were washed with 1X Accell siRNA medium (supplemented with Non-Essential Amino Acids and L-Glutamine), plated at 10^6^/mL in Accell siRNA medium, and treated with 1 µM STAT5-targeting siRNA (E-005169-00-0010), JNK-targeting siRNA (E-003514-00-0010), Mcl-1-targeting siRNA (E-004501-00-0010), Bcl-x_L_-targeting siRNA (E-003458-00-0010), or non-targeting siRNA (D-001910-10-50) for 48 h. After incubation, cells were treated with TSLP for the indicated times and processed for downstream applications (see RT-qPCR, immunoblotting, and analysis of survival).

### 2.6. RT-quantitative PCR

Skin MCs (5 × 10^5^ cells/mL) were deprived of GF/serum for 16 h in minimal medium. After starvation, cells were incubated with TSLP for the indicated time points. RT-qPCR was performed as described, using primers described therein [14]. Briefly, total RNA was isolated using the Nucleo spin RNA Kit (Macherey-Nagel, Düren, Germany), and RT-qPCR was carried out with the Light Cycler (LC) Fast Start DNA Master SYBR^®^ Green kit (Roche Applied-Science, Basel, Switzerland).

The expression levels of the target gene were quantified relative to the expression of the reference gene *Cyclophilin B*, using the 2^−ΔΔCT^ method. The oligonucleotide primers (TIB Molbiol, Berlin, Germany) for *SCF* and *IL-33* were as follows:


**SCF**
ForwardGCGTGGACTATCTGCCGCCG
ReverseAGCGCTGCGATCCAGCACAAA
**IL-33**
ForwardTGTCAACAGCAGTCTACTGTGG
ReverseTGGACCCCTGATATACCAAAGG

### 2.7. Immunoblotting

Skin MCs (5 × 10^5^ cells/mL) were deprived of GF/serum for 16 h in minimal medium. To study the phosphorylation of signaling molecules, cells were incubated for a further 30 min with TSLP. As positive controls, we used a combination of SCF (10 ng/mL) and IL-33 (20 ng/mL) for pERK and pp38, and human mast cell line (HMC)-1 cells (3 × 10^5^ cells per lane), kindly provided by Dr. J.H. Butterfield [43], for pSTAT3. To examine Mcl-1 and Bcl-x_L_ protein expression, cells were incubated with (or without) TSLP for 2 h or 4 h. After incubation, MCs were lysed and separated through 12% sodium dodecyl sulfate polyacrylamide gel electrophoresis (SDS-PAGE) [36,39]. The proteins were then transferred to nitrocellulose membranes. The membranes were blocked with 1X casein blocking buffer (Sigma Aldrich, St. Louis, MO, USA) and incubated with primary antibodies against Mcl-1, Bcl-x_L_, phospho/total-ERK, phospho/total-p38, phospho/total-JNK, phospho/total-STAT3, and phospho/total-STAT5, as well as ß-actin and Cyclophilin B as loading controls (each diluted 1:1000) (all from Cell Signaling Technologies), overnight, and subsequently with (1:20,000 diluted) HRP (horseradish peroxidase)-conjugated secondary antibodies (Merck Millipore, Darmstadt, Germany) for 1.5 h, as described [14,31,32]. Finally, blots were developed, and bands visualized by a chemiluminescence assay (Weststar Ultra 2.0, Cyanagen, Bologna, Italy), according to the manufacturer’s instructions, and the bands were recorded using a detector for chemiluminescence (Fusion FX7 Spectra, Vilber Lourmat, Eberhardzell, Germany). Densitometric measurements were assessed by the software ImageJ (National Institutes of Health, Bethesda, MD, USA) and arbitrary values were determined by the following equation:

(4) $$\mathrm{Relative}\;\mathrm{target}\;\mathrm{expression}=\frac{{\mathrm{density}}_{\mathrm{target}}}{{\mathrm{density}}_{\mathrm{housekeeping}\;\mathrm{gene}}}.$$

### 2.8. Statistical Analysis

Results are reported as mean ± standard error of the mean (SEM). In Figure 1, Figure 3, Figure S4, Figure S5, Figure 5, and Figure S6d,f,h, the data were statistically analyzed by paired *t*-test. For normalized data, the Wilcoxon matched-pairs signed rank test was used (Figure 3). In Figure S2, Figure 4a,b, and Figure 6a–h, the One-way Anova test with Tukey’s post-test for multiple comparisons was used. *P*-values less than 0.05 were considered statistically significant. Data were analyzed with GraphPad Prism Version 6.01 Software (San Diego, CA, USA).

## 3. Results

### 3.1. TSLP Counters Apoptosis of Skin MCs upon Growth Factor Withdrawal

After having confirmed that the TSLP receptor is expressed at mRNA (not shown, see also Motakis et al. [44]) and protein level (Figure S1), we determined whether TSLP influences skin MC survival. MCs were treated with TSLP in minimal medium (without GF/serum) and analyzed for signs of apoptosis. As TSLP at 7.5 ng/mL significantly decreased the percentage of annexin V-positive cells (Figure S2), we selected this concentration for further experiments. In accordance with recommendations from the Nomenclature Committee on Cell Death (NCCD) [45], we applied different techniques, each measuring a certain aspect of the apoptotic process, to confirm the above findings. Caspase-3 activity declined by 42% when TSLP was added (35000 RLU with TSLP versus 60000 RLU in untreated cells), as depicted in Figure 1a. The green fluorescent YoPro^TM^-1 dye permeates the slightly porous membrane of apoptotic cells and thus enables the visualization of phosphatidylserine (P-ser)-independent membrane alterations indicative of apoptosis. Using this method, TSLP-treated MCs displayed 44% less YoPro^TM^-1 positivity (Figure 1b).

Addressing DNA degradation, a characteristic feature of apoptosis in its later stage [46], we found that the ratio of cells displaying DNA degradation dropped by more than 50% in the presence of TSLP (Figure 1c).

Together, TSLP protects human skin MCs from apoptosis, as evidenced by different readouts.

### 3.2. TSLP Triggers Activation of STAT5 and JNK

**Figure 2:**
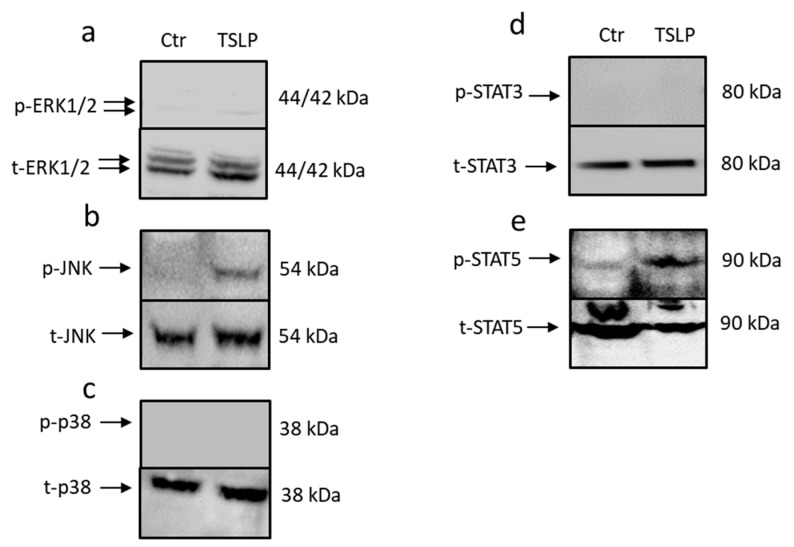
TSLP triggers the activation of signal transducer and activator of transcription (STAT)-5 and c-Jun-N-terminal kinase (JNK). The impact of TSLP treatment (at 7.5 ng/mL) on proximal signaling events after 30 min, as assessed by Western blotting using antibodies for (**a**) phospho (p) extracellular signal–regulated kinases (ERK)1/2 (n = 3), (**b**) pJNK, (**c**) pp38, (**d**) pSTAT3, and (**e**) pSTAT5 (shown are representative Western blots out of three independent experiments). The respective total (t) protein antibodies served as loading controls. Undetectable signals were negative also in time-course experiments.

Next, we addressed the downstream events triggered by TSLP in skin MCs. This was performed by studying the phosphorylation of STAT3, STAT5, and the three MAPKs JNK, ERK1/2, and p38. Both JNK and STAT5 were phosphorylated on the respective activatory sites following TSLP treatment (Figure 2a,e). This was verified by flow cytometry analysis, where the MFI increased by about 10-fold for p-STAT5 (Figure S4a) and by 4-fold for p-JNK (Figure S4b) in TSLP stimulated cells. In contrast, STAT3, ERK1/2, and p38 showed no modification (Figure 2a,c,d). Conversely, positive controls (MCs stimulated by IL-33/SCF [32] or HMC-1 cells [47]), which were used to confirm the quality of the technique, rendered the expected bands for all signaling intermediates (Figure S3).

### 3.3. MC Maintenance by TSLP Critically Depends on JNK and STAT5

**Figure 3:**
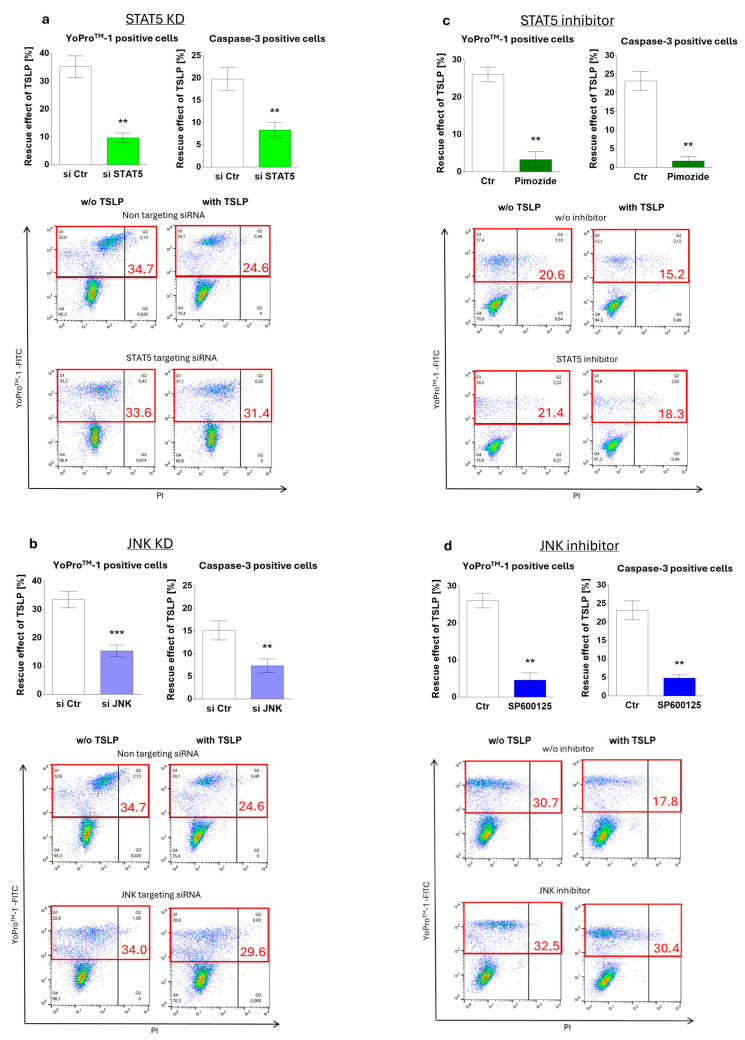
MC maintenance by TSLP critically depends on JNK and STAT5 activation. Impact of (**a**,**c**) STAT5 and (**b**,**d**) JNK perturbation on TSLP-promoted MC recovery (at 7.5 ng/mL) after 8 h, evaluated by the ratio of YoPro^TM^-1 positivity (corresponding to the percentage of early and late apoptotic/necrotic cells combined) in TSLP-treated versus untreated MCs (described in methods). (**a**,**b**) Interference by Accell^®^-mediated RNAi (48 h prior to TSLP treatment); (**c**,**d**) interference by specific inhibitors (STAT5 inhibitor: pimozide, JNK inhibitor: SP600125). Top: the results represent the mean ± SEM of six independent experiments. Bottom: representative flow cytometry dot plots (specified in red is the percentage of early and late apoptotic/necrotic cells combined); w/o—without. The data were analyzed by paired *t*-test, ** *p* < 0.01, *** *p* < 0.001.

Having identified JNK and STAT5 activation by TSLP, we investigated whether the two signaling intermediates were involved in survival maintenance. This was accomplished by two complementary strategies, namely pharmacological interference and knockdown (KD) of the respective component by RNA interference (RNAi). The latter approach revealed that survival promotion by TSLP indeed depends on STAT5, as the antiapoptotic effect decreased from 35% (control) to 10% after STAT5 KD, as assessed by YoPro^TM^-1 positivity (Figure 3a). The same tendency was found for caspase-3 activity (Figure 3a). Targeting JNK expression likewise affected TSLP-conferred apoptosis resistance, which declined from 35% to 15% (Figure 3b). For KD efficiency see Figure S5. Interestingly, the KD of STAT5 had no impact on the baseline survival of MCs in the absence of TSLP (Figure S6c), whereas JNK KD slightly increased the amount of YoPro^TM^-1 positive cells also without TSLP (Figure S6d).

Specific inhibitors supported the above findings. Pimozide (STAT5 inhibitor) led to a decrease from 34% to 4% (Figure 3c), while SP600125 (JNK inhibitor) diminished TSLP-mediated protection from 34% to 5% (Figure 3d). In line with their lacking activation by TSLP (Figure 2a,c), ERK1/2 and p38 (inhibited by SCH772984 and SB203580, respectively) were not involved in TSLP fostered survival (Figure S7).

Together, interference with STAT5 and JNK impeded TSLP from exerting its anti-apoptotic effect, implying key roles for these components in the antiapoptotic machinery contracted by TSLP.

### 3.4. TSLP up-Regulates Mcl-1 and Bcl-x_L_

**Figure 4:**
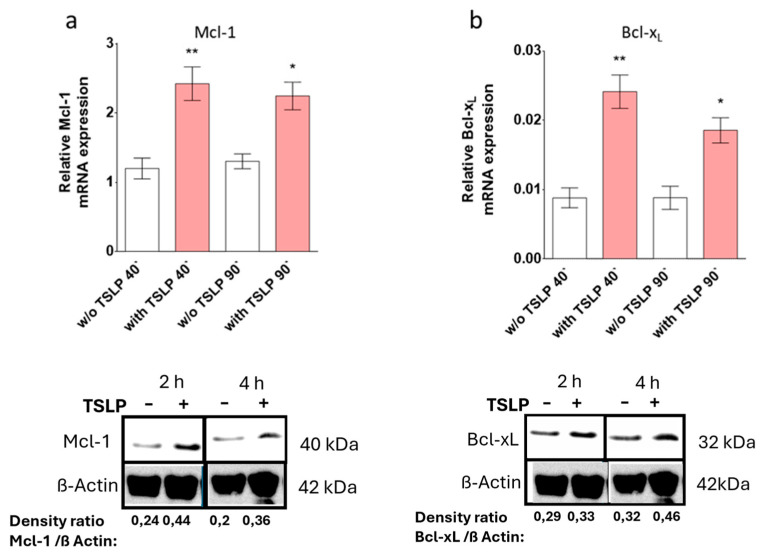
TSLP up-regulates Mcl-1 and Bcl-x_L_. TSLP-induced expression (at 7.5 ng/mL) was studied by (**a**,**b**) reverse transcription - quantitative polymerase chain reaction (RT-qPCR) analysis of (**a**) *Mcl-1* and (**b**) *Bcl-x_L_*; normalized to the housekeeping gene *Cyclophilin B*. The results represent the mean ± SEM of nine independent experiments. The data were analyzed by the one-way Anova test with Tukey’s post-test for multiple comparisons, comparing each treatment (40′ or 90′) with the respective control group; * *p* < 0.05, ** *p* < 0.01; and (**c**,**d**) Western blot analysis using the indicated antibodies (shown are representative Western blots out of three independent experiments); the anti-β-Actin antibody served as loading control. Densitometry arbitrary units were normalized to the housekeeping protein.

Various pro- and antiapoptotic factors are implicated in the orchestration of cell survival decisions, among which the Bcl-2 family is typically targeted by GFs. We delineated TSLP-mediated changes in Bcl-2 family members, finding significant increases in *Mcl-1* and *Bcl-x_L_* mRNA expression at both 40 and 90 min (Figure 4a,b). In contrast, TSLP treatment did not modulate the expression of *Bad*, *Bax*, *Bak*, *Bid*, and *Bcl-2* (Figure S8), although there was a slight tendency towards a reduced expression of proapoptotic *Bad* and *Bax* (Figure S8a,b).

Increased Mcl-1 and Bcl-x_L_ expression was verified by Western blot, whereby Bcl-x_L_ expression, and even more so Mcl-1 expression, were remarkably increased by TSLP, especially at the 2 h time point (Figure 4c,d).

### 3.5. Survival by TSLP Depends on Mcl-1 and Bcl-x_L_

As the above results suggested a role for Mcl-1, Bcl-x_L_ or both in survival promotion by TSLP, we employed an RNAi approach to experimentally prove this connection.

**Figure 5:**
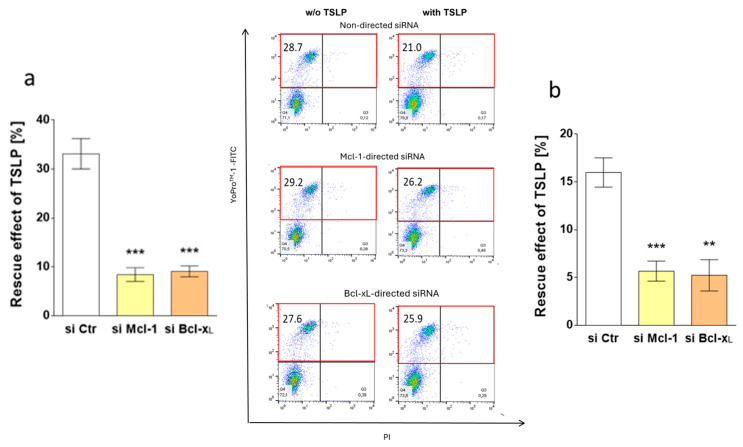
Survival prolongation by TSLP depends on Mcl-1 and Bcl-x_L_. Impact of Mcl-1 and Bcl-x_L_ knockdown on TSLP-promoted MC recovery (at 7.5 ng/mL), as evaluated by apoptosis reduction in TSLP-treated versus untreated MCs after 8 h. (**a**) Reduction of YoPro^TM^-1-positivity (corresponding to the percentage of early and late apoptotic/necrotic cells combined) as mean ± SEM of nine independent experiments (left) and representative flow cytometry dot plots (right) (specified in red is the percentage of early and late apoptotic/necrotic cells combined); w/o—without; (**b**) reduction of caspase-3 activity as mean ± SEM of nine independent experiments. The data were analyzed by paired *t*-test, ** *p* < 0.01, *** *p* < 0.001.

MC rescue by TSLP reached 16% using control siRNA, while the value equally dropped to 6% when Mcl-1- or Bcl-x_L_-targeting siRNA were used, as determined by caspase-3 activity (Figure 5b). This was verified by YoPro^TM^-1, whereby the protective effect of TSLP dropped from 30% (control) to 8% (Mcl-1-silencing) or 9% (Bcl-x_L_-silencing) (Figure 5a). In the absence of TSLP, we observed that the KD of Mcl-1 did not affect MC recovery (Figure S6a), while the deficiency of Bcl-x_L_ resulted in slightly reduced survival (Figure S6b). Taken together, both Mcl-1- and Bcl-x_L_ contribute comparably to the survival promotion conferred by TSLP.

### 3.6. STAT5 Perturbation Leads to Mcl-1 Downregulation, While Interference with JNK Attenuates Bcl-x_L_ Expression

**Figure 6:**
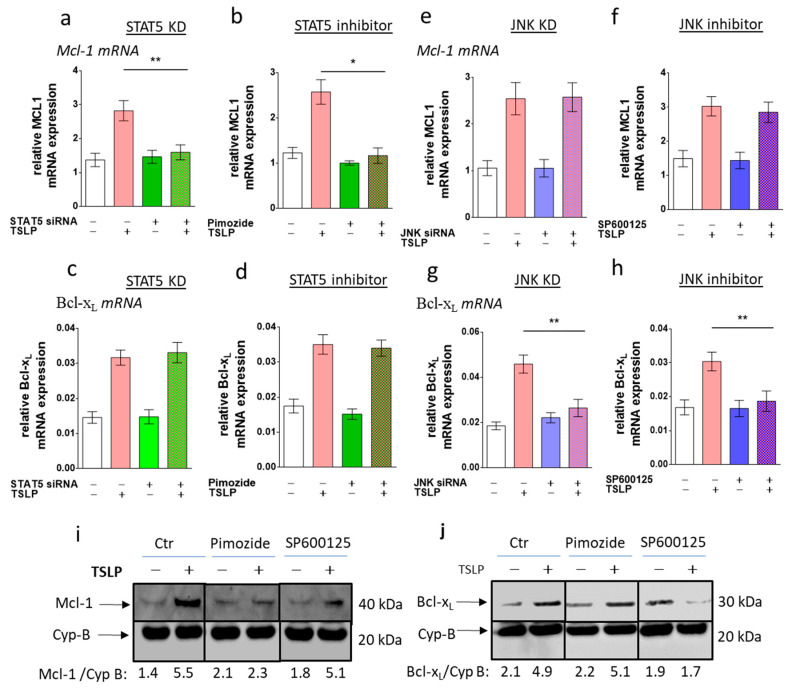
STAT5 perturbation leads to Mcl-1 downregulation, while interference with JNK attenuates Bcl-x_L_ expression. Impact of (**a**–**d**) STAT5 and (**e**–**h**) JNK perturbation on TSLP-triggered the expression (at 7.5 ng/mL) of (**a**,**b**,**e**,**f**) *Mcl-1* and (**c**,**d**,**g**,**h**) *Bcl-x_L_* after 40 min, using (**a**,**c**,**e**,**g**) knockdown by RNAi (48 h prior to the experiment), and (**b**,**d**,**f**,**h**) pre-incubation with specific inhibitors (STAT5 inhibitor: pimozide, JNK inhibitor: SP600125), evaluated by RT-qPCR analysis (normalized to *Cyclophilin B*). The results represent the mean ± SEM of five (RNAi) or six (inhibitors) independent experiments. The data were analyzed by the one-way Anova test with Tukey’s post-test for multiple comparisons, * *p* < 0.05, ** *p* < 0.01; (**i**,**j**) impact of specific inhibitors on the TSLP modulation of (**i**) Mcl-1 and (**j**) Bcl-x_L_ by Western blot analysis (shown are representative Western blots out of two independent experiments). Densitometry units were normalized to the housekeeping protein.

Our above findings demonstrated that STAT5 and JNK, as well as Mcl-1 and Bcl-x_L_, are required for survival prolongation by TSLP. We were finally interested in unraveling the relationships between the early and the later events. To this end, the TSLP modulation of Mcl-1 and Bcl-x_L_ levels was monitored upon interference with STAT5 and JNK function. While STAT5 perturbation completely blocked *Mcl-1* upregulation by TSLP (Figure 6a,b), the level of *Bcl-x_L_* was not affected by this treatment (Figure 6c,d). Again, the outcomes between STAT5-RNAi and the STAT5 inhibitor were consistent. Conversely, interference with the JNK function reversed TSLP’s effect on *Bcl-x_L_* expression (Figure 6g,h), whereas *Mcl-1* remained unaffected (Figure 6e,f). The nullified effect regarding *Bcl-x_L_* expression was consistent between RNAi and JNK inhibition. A clear distinction between the two antiapoptotic Bcl-2 members was confirmed at the protein level (Figure 6i,j). Conversely, the mRNA expression of *Bax* and *Bid*, studied for control purposes, was not affected by STAT5 or JNK interference (Figure S9a–h). We conclude that apoptosis resistance by TSLP results from the concomitant activation of a STAT5/Mcl-1 and a JNK/Bcl-x_L_ axis.

### 3.7. TSLP Protects Skin MCs Ex Vivo from Cell Death

**Figure 7:**
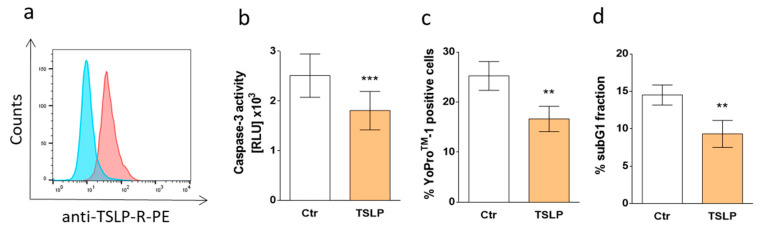
(**a**) Representative flow cytometry histogram of TSLP receptor (TSLPR) surface expression on skin MCs ex vivo (one representative out of four independent stainings is depicted)*;* blue: Isotype control; red: anti-TSLPR. (**b**–**d**) TSLP counters apoptosis of skin MCs ex vivo. Freshly isolated skin MCs were kept without or with TSLP (at 0.35 ng/mL) in serum/GF-free medium. (**b**) Caspase-3 activity as determined after 24 h by Caspase-Glo^®^ 3/7 assay; the results represent the mean ± SEM of six independent experiments. RLU = relative luminescence units (×10^3^). (**c**,**d**) percentage of cells with (**c**) YoPro^TM^-1 positivity (corresponding to the percentage of early and late apoptotic/necrotic cells combined) after 48 h or (**d**) fragmented DNA (PI staining, corresponding to the percentage of subG1 positive cells) after 48 h. The results represent the mean ± SEM of five independent experiments. The data were analyzed by paired *t*-test, ** *p* < 0.01, *** *p* < 0.001.

It was important to assess whether TSLP protects against MC apoptosis directly upon their isolation from skin tissue. In fact, several differences exist between skin MCs ex vivo and upon long-term pre-culture [38,44,48]. This also applies to the cells’ baseline survival, whereby ex vivo MCs display robust survival, even in the absence of exogenous GFs [14]. We treated skin-derived MCs immediately after purification with different concentrations of TSLP (in GF-/serum-free medium) and found full protection at the low concentration of 0.35 ng/mL (data not shown). To find the reason behind this exquisite sensitivity, we assessed TSLP receptor (TSLPR) expression by FACS and found robust staining in ex vivo MCs (Figure 7a), in accordance with the FANTOM5 atlas, where ex vivo skin MCs cells displayed the highest levels of the transcript across nearly 1800 samples (gene: *CRLF2*) [44,49].

We then confirmed that TSLP interferes with the cell death of ex vivo MCs. All apoptosis detection techniques applied (in analogy to 3.1) yielded congruent results, i.e., caspase-3 activity (very low in ex vivo MCs) was attenuated (Figure 7b), and so were the proportions of YoPro^TM^-1-positive cells (Figure 7c) and sub-G1 nuclei (Figure 7d). Considering the altered survival properties of ex vivo skin MCs, including their lower proneness to undergo cell death [14,38], the finding that low concentrations of TSLP can have a survival-favoring effect on these cells is notable and can potentially explain MC accumulation in the skin in TSLP-rich surroundings.

## 4. Discussion

Chronic inflammation is typically associated with increased numbers of MCs in the respective tissues, where the cells are believed to contribute to the underlying disease [1,2,4,50]. Therefore, there is strong interest in the mechanisms that govern MC death versus survival decisions, and MC apoptosis has been proposed as a viable means of limiting the aberrant MC function in allergic and chronic inflammation [9,51]. To be able to use this armamentarium in a targeted manner, it is essential to understand the mechanisms by which pathologic MC accumulation is brought about.

Here, we identified a new survival promoter of human skin MCs and revealed the mechanisms of its action. TSLP operates by the initial activation of STAT5 and JNK, which subsequently leads to Mcl-1 and Bcl-x_L_ upregulation, whose joint action finally impedes skin MC demise. Our results highlight the parallel existence of a STAT5–Mcl-1 and a JNK–Bcl-x_L_ axis in human skin MCs.

Together with IL-33, TSLP thus represents the second factor derived from resident skin cells (such as keratinocytes and fibroblasts [52,53]) to mediate the survival promotion of skin MCs in the absence of SCF [32]. Both cytokines may therefore orchestrate the numerical MC increase in the inflammatory micromilieu of skin disorders. It remains to be seen whether the two factors can further synergize with each other, or rather, display antagonistic actions in skin MCs. Of interest, IL-33 and TSLP overlap regarding JNK activation, while IL-33 lacks STAT5-inducing activity, but is highly efficient at p38 activation [31,32]. One may speculate that the antiapoptotic effect of TSLP is mediated by the induction of other MC-protective GFs, such as SCF or the presently mentioned IL-33. However, this is unlikely for several reasons. First, because of the rapidness of the effect, whereby Mcl-1 and Bcl-x_L_ were upregulated already at 40 min (Figure 4). Second, skin-derived MCs are poor producers of SCF and IL-33, as compared to other skin-resident cells, such as keratinocytes and fibroblasts (Figure S10), and this is perfectly in accordance with the FANTOM5 atlas, where both GFs were undetectable in the majority of MC samples [44]. Treating our MCs with TSLP did not change this picture, and the mRNA levels of *SCF* and *IL-33* remained unaffected and low (Figure S10). The antiapoptotic effect of TSLP was therefore likely direct without detour via another GF.

TSLP notably contributes to skin disorders like AD [20,54], and accordingly, binding to its specific receptor has been reported to regulate selected aspects of MC biology, including maturation and mediator secretion [24,25,26,27]. In this regard, Lai et al. demonstrated that TSLP can increase Tryptase storage [55], whereas Rönnberg et al. showed an opposite effect [28], underlining the heterogeneous character of MCs. Han et al. described an antiapoptotic effect in the human MC line (HMC)-1 (as well as in the immature murine bone marrow-derived mast cell (BMMC) [27], while human lung MCs did not experience survival promotion by TSLP [25]. Together, this indicates that immaturity and/or malignant transformation may have pre-disposed to survival rescue by TSLP, which is also in accordance with a recent finding of increased TSLPR expression in the continuously growing MC line ROSA vis-à-vis primary MCs [28]. However, because skin MCs, not studied previously, represent primary, fully differentiated MCs, just like lung MCs, our current data instead imply that MCs at a post-maturation stage are amenable to survival protection by TSLP and that the microenvironment in which they completed maturation will primarily dictate this response. Because skin MCs are of the so-called MC_TC_ subset, whereas most MCs in the lung are of the MC_T_ type [29,30], the current results may also suggest a dichotomy between MC_TC_ and MC_T_ MCs in their response pattern to TSLP. The TSLP/TSLPR axis has been indicated to strengthen the MC compartment also in vivo in humans and mice [27,55].

Our findings with freshly isolated dermal MCs (Figure 7) strengthen the assumption that MCs are protected by TSLP in the skin habitat. In fact, the cytokine considerably reduces apoptosis in this physiological MC subtype, despite the low proneness of these cells to undergo cell death, even under harsh conditions. Interestingly, TSLP is efficient at low concentrations, i.e., within the range produced by primary keratinocytes [41], which is likely related to the cells’ intense expression of TSLPR (Figure 7a). Although inter-cell comparisons based on FACS measurements may be somewhat problematic, tendencies can be inferred, at least for the percentages of receptor-positive cells. This limitation in mind, ex vivo skin MCs seem to express TSLPR at higher levels compared to cultured skin MCs (see Figure 7a versus Figure S1) and in comparison to primary lung-derived MCs [28]. The difference at mRNA level (ex vivo versus cultured) is, moreover, in accordance with the FANTOM5 atlas [44,49]. Of note, the protective effect of TSLP in ex vivo MCs was in a similar range as the one afforded by SCF in these cells [14]. Therefore, both cultured and ex vivo skin MCs respond similarly to TSLP with regard to survival promotion. Since cultured MCs depend more strongly on exogenous GFs and are overall more prone to die, these cells were more adequate to study the molecular underpinnings behind TSLP’s protection.

Addressing the mechanisms, we first found that STAT5 and JNK are both phosphorylated and implicated in TSLP-conferred apoptosis resistance, as their ablation or inhibition impaired survival promotion.

Although TSLP can trigger disparate signal transduction pathways, depending on the cell type [56], the activation of STAT5 represents the canonical and best documented pathway in a number of cells [57,58,59,60,61,62,63]. Its activation in MCs was therefore plausible, although this had not been addressed by previous studies. Surprisingly, STAT5 activation was complemented by JNK, which proved to be a further effector pathway organizing MC survival, as its inhibition or KD abolished TSLP’s survival-promotion. In contrast to STAT5, JNK has, to our knowledge, only been reported as being triggered by TSLP in stromal, but not in hematopoietic cells [64].

Our finding that STAT5 contributes to TSLP’s survival machinery in skin MCs is consistent with a long series of studies, demonstrating its relevance in the MC lineage [65,66,67,68,69,70]. In fact, STAT5 can contribute to the expression of antiapoptotic Bcl-2 members in MCs, even if not specifically Mcl-1, but this distinction may stem from the different MC subsets used in their (murine BMMCs) [65] vis-à-vis our study.

In contrast, JNK is described as a “double-edged sword”, either promoting or inhibiting apoptosis, depending on the precise conditions [71,72]. In skin MCs a survival-prolonging effect seems to prevail, as observed for TSLP treated MCs but also somewhat at baseline, as interference with JNK acted in a proapoptotic manner under both conditions (Figure 3b,d, Figure S6d,h). This is in accordance with a previous report showing that survival promotion by KIT occurs in a JNK-dependent manner in HMC-1 cells [73].

We then addressed the potential downstream targets of STAT5 and JNK. The Bcl-2 family, comprising antiapoptotic members like Bcl-2, Bcl-x_L_, Mcl-1, and proapoptotic entities, including Bad, Bax, Bak, and Bid, crucially controls the mitochondrial pathway of apoptosis. Their participation in the regulation of MC survival has been described in multiple reports [14,74,75,76].

We found that Bcl-x_L_ and Mcl-1 were distinctively upregulated by TSLP. To clarify their individual roles in survival promotion, we employed RNA interference to deplete Mcl-1 and Bcl-x_L_ expression prior to stimulation with TSLP. Interestingly, Mcl-1 and Bcl-x_L_ both equally imparted TSLP protection. This is in line with previous reports demonstrating that Mcl-1 is tightly connected to death/survival decisions of the lineage in transgenic mice [77], and in MCs of murine [78] and human origin [14,76,79]. In fact, we recently identified Mcl-1 as the main contributor to the longevity of human skin MCs [14], a function supported by the vast expression of Mcl-1 in ex vivo skin MCs [44]. Upon culture, the levels of Mcl-1 decline, and this reduced expression is insufficient to maintain survival unless further supported by GFs. Consistently, we found here that the KD of Mcl-1 did not impair baseline survival, suggesting that Mcl-1 is required to execute the protective function of TSLP, whereas it does not seem crucial for survival control in its absence.

KD experiments identified Bcl-x_L_ as another factor controlling MC survival in the presence of TSLP. This antiapoptotic molecule has likewise been widely documented as a survival promoter of MCs, however primarily in response to IL-33, SCF, and IgE receptor activation [16,74,75,76]. Conversely, its involvement in TSLP-mediated responses was unexpected and represents a novel finding of this study.

After revealing the crucial roles of Mcl-1 and Bcl-x_L_, we sought to examine potential links between the proximal events (STAT5 and JNK activation) and the subsequent upregulation of these factors. Intriguingly, we found a clear-cut distinction between the Bcl-2 family members, as inhibition of STAT5 counteracted Mcl-1 with no impact on Bcl-x_L_ expression, whereas JNK perturbation resulted in decreased Bcl-x_L_ upregulation without affecting Mcl-1 expression, speaking in favor of well separated pathways. In fact, Mcl-1 can be activated by STAT5 and regulate survival in different cells [80,81,82]. Because Bcl-x_L_ has also been reported as a target of STAT5 in other cell types [83,84], the precise disconnection in the transcriptional prerequisites uncovered here was unexpected. In fact, Bcl-x_L_ was even found to be under negative influence from JNK in a number of other cells [85,86].

We assume that the simultaneous upregulation of both factors is necessary to exceed a certain “antiapoptotic” threshold to allow for TSLP’s protective function to come into force. In this scenario, TSLP would only counteract cell death in the event of both antiapoptotic Bcl-2 members jointly reaching this point. This theory is supported by findings showing that mitochondrial membrane disruption and concomitant cytochrome c release can depend on a distinct threshold of mitochondrial membrane permeabilization [87].

## 5. Conclusions

Taken together, we uncovered TSLP as a novel survival factor of human skin MCs, which exerts its antiapoptotic effect by the synchronized activation of STAT5 and JNK, the ensuing segregated upregulation of Mcl-1 and Bcl-x_L_, and consequent apoptosis resistance. The increased lifespan is thus owed to the concomitant activation of two axes, namely STAT5/Mcl-1 and JNK/Bcl-x_L_. Our findings support a role for TSLP as a crucial component of the MC supportive niche in lesional skin, and its overabundance during disturbed homeostasis may help explain MC accumulation in cutaneous pathology.

## Supplementary Materials

The following are available online at https://www.mdpi.com/2073-4409/8/8/829/s1, Figure S1: Representative flow cytometry histogram of TSLP-R surface expression on cultured skin MCs. Figure S2: TSLP dose-dependently inhibits phosphatidylserine externalization in GF-deprived MCs. Figure S3: STAT3 is activated in HMC-1 cells, and in contrast to TSLP, the combination of SCF and IL-33 triggers phosphorylation of ERK1/2 and p38. Figure S4: Phosphorylation of STAT5 and JNK by TSLP by flow cytometry. Figure S5: Knockdown efficiency of STAT5 and JNK. Figure S6: Influence of Mcl-1, Bcl-xL, STAT5 and JNK on MC survival in the absence of TSLP. Figure S7: ERK1/2 and p38 are not implicated in TSLP-induced survival rescue. Figure S8: TSLP does not affect the expression of several Bcl-2 family members. Figure S9: Perturbation of STAT5 and JNK activity leaves expression of Bid and Bax unaffected. Figure S10: Basal and TSLP-treated human skin MCs are poor producers of SCF and IL-33 in comparison to keratinocytes and fibroblasts—no regulation by TSLP.
