# Correction: Hazzan et al. Thymic Stromal Lymphopoietin Interferes with the Apoptosis of Human Skin Mast Cells by a Dual Strategy Involving STAT5/Mcl-1 and JNK/Bcl-x_L_. *Cells* 2019, *8*, 829

**DOI:** 10.3390/cells13242105

**Published:** 2024-12-19

**Authors:** Tarek Hazzan, Jürgen Eberle, Margitta Worm, Magda Babina

**Affiliations:** Department of Dermatology, Venerology and Allergy, Charité—Universitätsmedizin Berlin, Charitéplatz 1, 10117 Berlin, Germany

**Error in Figure 3**

In the original publication [1], there was a mistake in the published version of Figure 3a.

Dot plots from the same measurements were mistakenly used for different treatments. In Figure 3a, the treatments “w/o TSLP, non-targeting” and “w/o TSLP, STAT5-targeting” as well as “with TSLP, non-targeting” and “with TSLP, STAT5-targeting” originated from the same measurement.

In the original publication, there was also a mistake in the published version of Figure 3b.

The same error occurred here as described for Figure 3a; dot plots were assembled based on the same measurements and were mistakenly used to represent different treatments. In Figure 3b, the treatments “w/o TSLP, STAT5-targeting” (from Figure 3a) and “w/o TSLP, JNK-targeting” (from Figure 3b) as well as “with TSLP, STAT5-targeting” (from Figure 3a) and “with TSLP, JNK-targeting” (from Figure 3b) were apparently derived from the same measurement.

The same error occurred in Figure 3c; the same dot plots were mistakenly used for different treatments. The images “w/o TSLP, w/o STAT5-Inhibitor” and “w/o TSLP, with STAT5-Inhibitor” originated from the same measurement. This results in a noticeable similarity between the dot patterns.

The corrected version of Figure 3 appears below.

The original figure legend remains in place.

**Figure 3:**
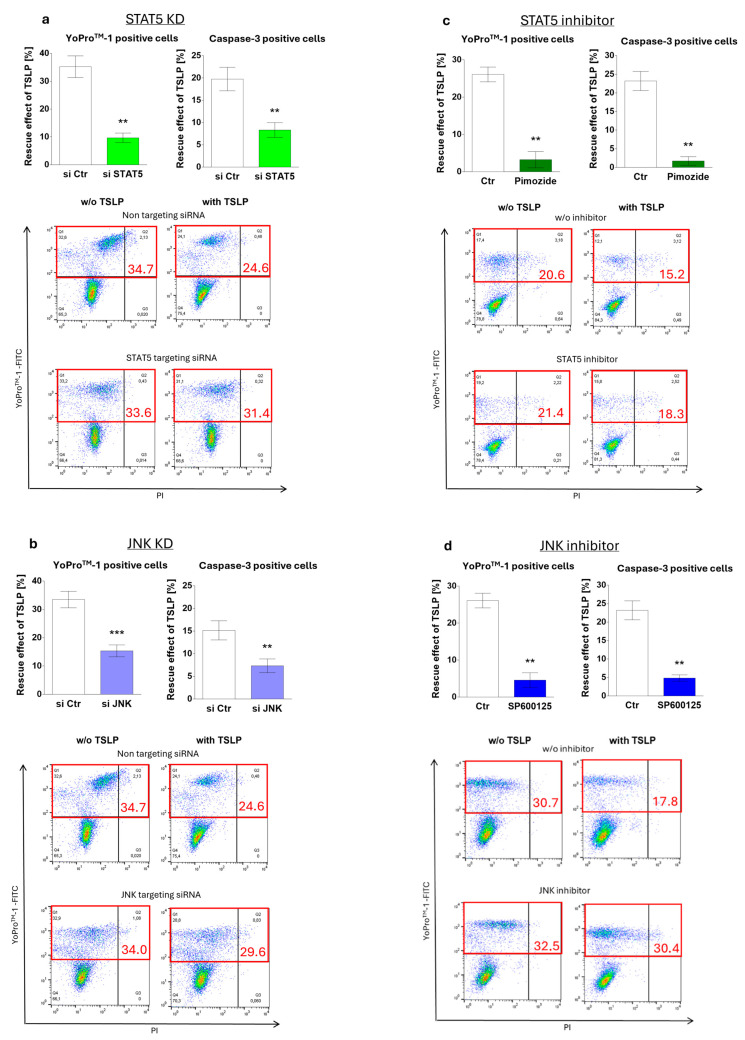
MC maintenance by TSLP critically depends on JNK and STAT5 activation. Impact of (**a**,**c**) STAT5 and (**b**,**d**) JNK perturbation on TSLP-promoted MC recovery (at 7.5 ng/mL) after 8 h, evaluated by the ratio of YoPro^TM^-1 positivity (corresponding to the percentage of early and late apoptotic/necrotic cells combined) in TSLP-treated versus untreated MCs (described in methods). (**a**,**b**) Interference by Accell^®^-mediated RNAi (48 h prior to TSLP treatment); (**c**,**d**) interference by specific inhibitors (STAT5 inhibitor: pimozide, JNK inhibitor: SP600125). Top: the results represent the mean ± SEM of six independent experiments. Bottom: representative flow cytometry dot plots (specified in red is the percentage of early and late apoptotic/necrotic cells combined); w/o—without. The data were analyzed by paired *t*-test, ** *p* < 0.01, *** *p* < 0.001.

**Error in Figure 4**

In the original publication, there was a mistake in the published versions of Figure 4c,d.

A pipetting error (the samples were applied in the wrong order, with 4 h first and then 2 h) contributed to the erroneous preparation of the images (rotation and shifting of the b-actin blot). In addition, membranes were mixed up, resulting in the use of non-corresponding ones for the target protein and the housekeeping protein.

Upon detailed inspection of all blots, these issues could be corrected.

The corrected version of Figure 4 appears below. 

The original figure legend remains in place.

**Figure 4:**
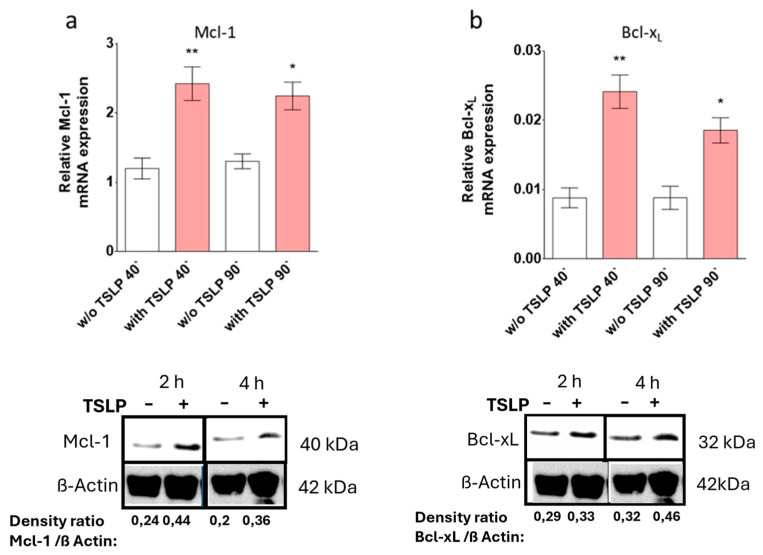
TSLP up-regulates Mcl-1 and Bcl-x_L_. TSLP-induced expression (at 7.5 ng/mL) was studied by (**a**,**b**) reverse transcription - quantitative polymerase chain reaction (RT-qPCR) analysis of (**a**) *Mcl-1* and (**b**) *Bcl-x_L_*; normalized to the housekeeping gene *Cyclophilin B*. The results represent the mean ± SEM of nine independent experiments. The data were analyzed by the one-way Anova test with Tukey’s post-test for multiple comparisons, comparing each treatment (40′ or 90′) with the respective control group; * *p* < 0.05, ** *p* < 0.01; and (**c**,**d**) Western blot analysis using the indicated antibodies (shown are representative Western blots out of three independent experiments); the anti-β-Actin antibody served as loading control. Densitometry arbitrary units were normalized to the housekeeping protein.

**Error in Figure 5**

In the original publication, there was a mistake in the published version of Figure 5a.

The same error occurred here as described above for Figure 3a–c; the same measurements were mistakenly used to compile dot plots representing different treatments, such that in Figure 5a, the treatments “w/o TSLP, Mcl-1 targeting” and “with TSLP, Mcl-1 targeting” as well as “w/o TSLP, Bcl-xL targeting” and “with TSLP, Bcl-xL targeting” originated from the same measurement.

The corrected version of Figure 5 appears below. 

The original figure legend remains in place.

**Figure 5:**
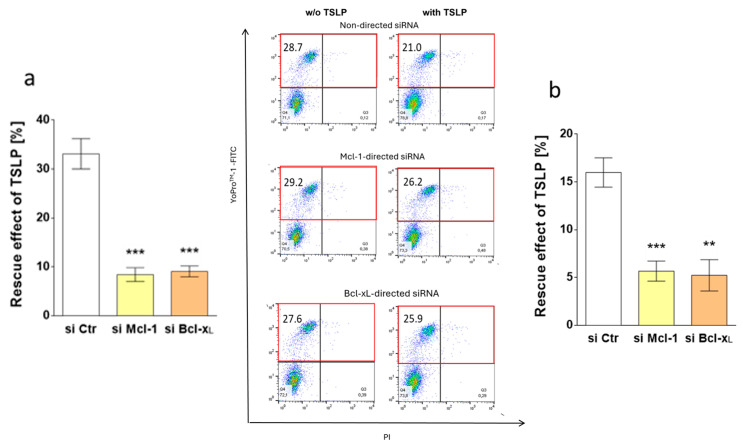
Survival prolongation by TSLP depends on Mcl-1 and Bcl-x_L_. Impact of Mcl-1 and Bcl-x_L_ knockdown on TSLP-promoted MC recovery (at 7.5 ng/mL), as evaluated by apoptosis reduction in TSLP-treated versus untreated MCs after 8 h. (**a**) Reduction of YoPro^TM^-1-positivity (corresponding to the percentage of early and late apoptotic/necrotic cells combined) as mean ± SEM of nine independent experiments (left) and representative flow cytometry dot plots (right) (specified in red is the percentage of early and late apoptotic/necrotic cells combined); w/o—without; (**b**) reduction of caspase-3 activity as mean ± SEM of nine independent experiments. The data were analyzed by paired *t*-test, ** *p* < 0.01, *** *p* < 0.001.

The authors state that the scientific conclusions are unaffected. This correction was approved by the Academic Editor. The original publication has also been updated.
